# Role of A Novel Angiogenesis FKBPL-CD44 Pathway in Preeclampsia Risk Stratification and Mesenchymal Stem Cell Treatment

**DOI:** 10.1210/clinem/dgaa403

**Published:** 2020-07-03

**Authors:** Naomi Todd, Ross McNally, Abdelrahim Alqudah, Djurdja Jerotic, Sonja Suvakov, Danilo Obradovic, Denise Hoch, Jose R Hombrebueno, Guillermo Lopez Campos, Chris J Watson, Miroslava Gojnic-Dugalic, Tatjana P Simic, Anna Krasnodembskaya, Gernot Desoye, Kelly-Ann Eastwood, Alyson J Hunter, Valerie A Holmes, David R McCance, Ian S Young, David J Grieve, Louise C Kenny, Vesna D Garovic, Tracy Robson, Lana McClements

**Affiliations:** 1 The Wellcome-Wolfson Institute for Experimental Medicine, School of Medicine, Dentistry and Biomedical Sciences, Queen’s University Belfast, Northern Ireland, UK; 2 The School of Pharmacy, The Hashemite University, Amman, Jordan; 3 Medical Faculty, University of Belgrade, Belgrade, Serbia; 4 Department of Nephrology and Hypertension, Mayo Clinic, Rochester, MN, US; 5 Department of Gynaecology and Obstetrics, Medical University Graz, Graz, Austria; 6 Centre for Public Health, School of Medicine, Dentistry and Biomedical Sciences, Queen’s University Belfast, Northern Ireland, UK; 7 Royal Jubilee Maternity Hospital, Belfast Health and Social Care Trust, Northern Ireland, UK; 8 Royal Victoria Hospital, Belfast Health and Social Care Trust, Northern Ireland, UK; 9 The Irish Centre for Foetal and Neonatal Translational Research (INFANT) and Department of Obstetrics and Gynaecology, University College Cork, Cork, Republic of Ireland; 10 Department of Women’s and Children’s Health, Institute of Translational Research, Faculty of Health and Life Sciences, University of Liverpool, Liverpool, UK; 11 School of Pharmacy and Biomolecular Sciences, Irish Centre for Vascular Biology, Royal College of Surgeons in Ireland (RCSI), Dublin, Republic of Ireland; 12 School of Life Sciences, Faculty of Science, University of Technology Sydney, NSW, Australia

**Keywords:** preeclampsia, angiogenesis, risk prediction, mesenchymal stem cell treatment, trophoblast cells, endothelial function

## Abstract

**Context:**

Preeclampsia is a leading cardiovascular complication in pregnancy lacking effective diagnostic and treatment strategies.

**Objective:**

To investigate the diagnostic and therapeutic target potential of the angiogenesis proteins, FK506-binding protein like (FKBPL) and CD44.

**Design and Intervention:**

FKBPL and CD44 plasma concentration or placental expression were determined in women pre- or postdiagnosis of preeclampsia. Trophoblast and endothelial cell function was assessed following mesenchymal stem cell (MSC) treatment and in the context of FKBPL signaling.

**Settings and Participants:**

Human samples prediagnosis (15 and 20 weeks of gestation; n ≥ 57), or postdiagnosis (n = 18 for plasma; n = 4 for placenta) of preeclampsia were used to determine FKBPL and CD44 levels, compared to healthy controls. Trophoblast or endothelial cells were exposed to low/high oxygen, and treated with MSC-conditioned media (MSC-CM) or a FKBPL overexpression plasmid.

**Main Outcome Measures:**

Preeclampsia risk stratification and diagnostic potential of FKBPL and CD44 were investigated. MSC treatment effects and FKBPL-CD44 signaling in trophoblast and endothelial cells were assessed.

**Results:**

The CD44/FKBPL ratio was reduced in placenta and plasma following clinical diagnosis of preeclampsia. At 20 weeks of gestation, a high plasma CD44/FKBPL ratio was independently associated with the 2.3-fold increased risk of preeclampsia (odds ratio = 2.3, 95% confidence interval [CI] 1.03-5.23, *P* = 0.04). In combination with high mean arterial blood pressure (>82.5 mmHg), the risk further increased to 3.9-fold (95% CI 1.30-11.84, *P* = 0.016). Both hypoxia and MSC-based therapy inhibited FKBPL-CD44 signaling, enhancing cell angiogenesis.

**Conclusions:**

The FKBPL-CD44 pathway appears to have a central role in the pathogenesis of preeclampsia, showing promising utilities for early diagnostic and therapeutic purposes.

Hypertensive disorders of pregnancy are a leading cause of maternal and neonatal morbidity and mortality (1). Preeclampsia, one of the most dangerous hypertensive disorders of pregnancy, develops after 20 weeks of gestation and occurs in 2% to 8% of pregnancies (2). Preeclampsia is characterized by the new onset of high blood pressure (≥140/90 mm Hg) on 2 separate occasions and evidence of end-organ involvement, most commonly renal or liver, in pregnancy (3). There are 2 main phenotypes of preeclampsia: (i) early-onset or preterm preeclampsia diagnosed prior to 34 weeks of gestation and (ii) late-onset or term preeclampsia diagnosed from 34 weeks of gestation onward (4,5). There is great interest in developing biomarkers of preeclampsia, which can predict likelihood of, or evolving, preeclampsia from the first to third trimester (6). However, poorly understood molecular regulation of pathogenesis has hindered the development of reliable biomarkers especially in relation to the late-onset and evolving preeclampsia (6). Clinically employed screening tests based on angiogenesis-related biomarkers (eg, soluble fms-like tyrosine kinase 1 [sFlt-1] and placental growth factor [PlGF]) are able to predict the onset of preeclampsia only a few weeks before presentation of clinically obvious preeclampsia (7). Other predictive algorithms have been used successfully early in pregnancy between 11 and 13 weeks of gestation based on maternal characteristics, uterine artery pulsatility index and angiogenic factors such as PlGF and PAPP-A (8). However, these are mainly effective at predicting early-onset or preterm preeclampsia. Therefore, risk stratification strategies for late-onset preeclampsia as well as evolving preeclampsia between the second and third trimester of pregnancy, are lacking. This is particularly important as stratifying pregnancies at risk of preeclampsia has been associated with improved pregnancy outcomes (9) and appears cost-effective, even a few weeks before the clinical onset of disease (10).

Although low-dose aspirin has been effective at preventing early-onset preeclampsia (11), the only treatment of established preeclampsia is delivery of the placenta, which necessitates delivery of the baby, frequently at preterm gestations. Mesenchymal stem cells (MSCs) are emerging as an attractive treatment in various diseases and injury models due to their low immunogenicity, easy cultivation, and expansion in vitro. In preclinical models of preeclampsia, MSCs demonstrated therapeutic efficacy (12,13), likely through paracrine signaling; however, the underlying mechanisms are not yet well understood (14,15).

FK506-binding protein like (FKBPL) is a peptidyl prolyl cis trans isomerase, which belongs to the immunophilin protein group with important roles in vascular diseases (16). FKBPL has a potent anti-angiogenic activity, with the high levels secreted from endothelial cells (17). As such, FKBPL plays an important role in both developmental and pathological angiogenesis; FKBPL knockout mice were embryonically lethal whereas FKBPL heterozygous knockdown mice showed signs of impaired vascular integrity (18). Furthermore, FKBPL also appears to regulate “stemness”; with high levels promoting differentiation, while low levels increased stem cell–like characteristics (19-21). Inhibition of angiogenesis and stem cell signaling by FKBPL appears to be dependent on CD44; knockdown of CD44 abrogates the FKBPL-mediated inhibition of angiogenesis and binding assays suggest an interaction between the 2 proteins (17,22). There is also evidence for targeting the signal transducer and activator of transcription 3–nuclear factor kappa B inflammatory pathway (20). The potential of FKBPL as a prognostic biomarker has been previously discussed in relation to other pathologies such as cancer (23-25), but not in the context of preeclampsia.

As a critical inhibitor of angiogenesis, we hypothesized that the FKBPL-CD44 pathway has an important role in the pathogenesis of preeclampsia, which could be utilized for diagnostic and therapeutic purposes. To address this hypothesis, we demonstrated that FKBPL is differentially secreted early in pregnancy (weeks 15 and 20 of gestation) in women who proceeded to develop preeclampsia. At 20 weeks of gestation, the CD44/FKBPL ratio in combination with mean arterial blood pressure (MAP) was capable of predicting a four-fold increased risk of preeclampsia in nulliparous pregnant women; most of the pregnancies in our cohort proceeded to develop late-onset preeclampsia. The CD44/FKBPL ratio was also capable of diagnosing preeclampsia and was differentially expressed in placentae affected by preeclampsia. Further studies indicated that FKBPL regulates trophoblast and endothelial cell function important for spiral uterine artery (SUA) development. Hypoxia and MSCs are capable of inhibiting the FKBPL-CD44 anti-angiogenic pathway.

## Materials and Methods

### Enzyme-linked immunosorbent assay of human plasma and isolated MSCs

Ethylenediamine tetra-acetate plasma samples obtained from whole blood were centrifuged for 15 min at 2000 rpm at 4°C. Upon separation, plasma was stored at −80°C until further usage. For CD44, the inter-assay coefficient of variability (CV) was 6.9% and the average intra-assay CV % was 3.9%. The average intra- and inter-assay CV for FKBPL was 5.5% and 17.1%, respectively. Samples were assayed in duplicates and only included if CV percentage was below 15. Even though the inter-assay CV for FKBPL was high, we ensured that the investigators were blinded to the identity of the samples, and on each plate we had similar numbers of preeclampsia and control samples at each time point. Each enzyme-linked immunosorbent assay plate included a randomized selection of preeclampsia and control samples labeled with unique identifiable codes provided by the biobank, which were blinded to both operators. Plasma was diluted 2-fold for FKBPL (Cloud-Clone Corp., USA) or 40-fold for CD44 (Abcam, UK) using standard diluent provided by the kit. Human MSCs were isolated from abdominal fat tissue removed from women during C-section and cultured for 48 h as described previously (26) before medium was collected and secreted FKBPL measured. Human plasma samples used were from two separate cohorts of pregnant women with established clinical preeclampsia and normotensive controls, pregnant women with prediagnosis of clinical preeclampsia collected at 15 and 20 weeks of gestation, and healthy controls of the same gestational age. All studies with human samples were approved by the local institutional review boards and written informed consent was obtained from all participants.

### Immunofluorescence staining of placental tissue

Women considered to be at high risk of preeclampsia and their matched controls provided written informed consent at the Royal Jubilee Maternity Hospital (27). Ethical approval was obtained from the National Health Service Health Research Authority (ORECNI, 14/NI/1068). Ethical approval was also obtained from the School of Medicine, Dentistry and Biomedical Sciences (Queen’s University Belfast). Following delivery, each placenta was collected and processed by the Northern Ireland Biobank (28). Preeclampsia samples (n = 4) were paired 1-to-1 with normotensive control samples (n = 4) based on age and body mass index (BMI). The samples were also matched for gestational age. Placental slides for immunofluorescence analysis were prepared using fresh frozen placental tissue near the umbilical cord and stained for FKBPL (Cat. no.: 10060-1-AP; Proteintech, UK) and CD31 (Cat. no: 351-004; Synaptic Systems, Germany). For the immunofluorescence analysis, 4 random fields of view were selected to evaluate the mean fluorescence intensity in each sample/slide and quantify protein expression as previously described (29). Background was acquired from a vacant area of the labeled section and subtracted from the raw images to eliminate background noise.

### Cell culture

A total of 5 cell lines were used in subsequent investigations. All cells were initially grown in monolayers in vented 75 cm^2^ tissue culture flasks (Nunc; Thermoscientific, Denmark) at 37°C, in an atmosphere of 5% CO_2_ and 95% air (Laboratory Technical Engineering, UK). All cell lines were authenticated by short tandem repeat profiling performed by the suppliers and routinely tested for Mycoplasma.

1. Human placenta choriocarcinoma (BeWo) (ATCC, USA) were maintained in DMEM/F12 (ThermoFisher, UK) medium supplemented with 10% fetal bovine serum (FBS) (ThermoFisher, UK).
2. Human placenta choriocarcinoma (Jar) (ATCC, USA) were maintained in RPMI-1640 medium supplemented with 10% FBS.
3. Human umbilical vein endothelial cells (HUVECs) (ATCC, USA) were maintained in endothelial cell growth medium MV2 (Promocell, Germany), supplemented with low serum growth supplement containing the following: 5% fetal calf serum, epidermal growth factor 5 ng/mL, basic fibroblast growth factor 10 ng/mL, insulin-like growth factor 20 ng/mL, vascular endothelial growth factor 0.5 ng/mL, ascorbic acid 1 μg/mL and hydrocortisone 0.2 μg/mL, on gelatine-coated tissue culture flasks.
4. ACH-3P cells were kindly donated by Professor Gernot Desoye (Graz Medical University, Austria). Briefly, immortalized AC1-1 cells and primary trophoblast cells were fused as previously described (30) to form a unique ACH-3P cell line. Immortalized first-trimester cells were maintained in Hams F-12 medium (Thermofisher, UK) supplemented with 10% FBS. All experiments with ACH-3P cells were carried out at the Graz Medical University.
5. Primary human bone marrow–derived MSCs, which met the criteria of the International Society for Cellular Therapy (31), were obtained from the Institute for Regenerative Medicine at Texas A&M Health Science Center, Baylor, Scott and White Hospital (Temple, Texas, USA). MSCs were maintained in αMEM (Thermo Fisher Scientific, UK) supplemented with 16.5% heat-inactivated FBS, 50 µg/mL penicillin-streptomycin , and 4 mM L-glutamine (Thermo Fisher Scientific, UK) and used up to passage 7 as previously described (32).

### Treatment with MSCs

MSCs were seeded in 6 well plates in prescribed supplemented media for HUVEC, BeWo, and Jar at a ratio of 1 MSC to every 5 HUVEC, BeWo, or Jar. The cells were allowed to adhere and conditioned in the prescribed media for 24 h under 21% O_2_ conditions. Prior to the day of experimentation, the conditioned medium was aspirated off, collected, and centrifuged for 5 min at 1200 rpm to remove cell debris. Conditioned media was then pre-equilibrated to 21% (normoxia) or 1% (hypoxia) O_2_ prior to being used for other downstream experiments.

### FKBPL plasmid overexpression

Cells were grown to 50% (HUVECs) or 60% to 70% (BeWo or Jar) confluence before being transiently transfected with 1µg FKBPL plasmid (Sino Biological, PA, USA), or equivalent amounts of control pCMV plasmid (Sino Biological, USA) using EndoFectin (GeneCopoeia, USA). Following a 24-h transfection, the cells were trypsinized and reseeded for the tubule formation assay. Protein lysates were also collected following 24 h of transfection and subjected to Western blotting analysis.

### Clonogenic assay

To assess the ability of BeWo and Jar to form clones, 1000 and 2000 cells were seeded in 6 well plates, incubated for 16 to 18 days in 21% or 1% O_2_. Following incubation, media were aspirated and colonies washed prior to being stained with 0.4% crystal violet. Colonies containing 50 cells or more were manually counted and identified as “holoclones,” “meroclones,” or “paraclones” according to their morphologies using a DMI8 inverted light microscope.

### Tubule formation assay

To assess the ability of HUVECs to form tube-like structures, 7×10^5^ HUVEC were resuspended in 500 μL MSC-CM, or control media, and stained with calcein (2µg/mL) (Thermo Fisher Scientific, UK) prior to being seeded on phenol red free reduced-growth factor Matrigel under 21% and 1% O_2_ for 6 h. Tubule formation was imaged by randomly capturing 6 images per well using a DMI8 inverted florescence microscope. Total tube length was quantified using Image J software (NIH, USA).

### Wound scratch migration assay

To investigate the effects of MSC conditioned media on trophoblast and endothelial wound closure under 21% and 1% O_2_, a wound scratch migration assay was set up as described before (32). HUVECs and BeWo were seeded at a cell density of 5×10^4^ cells, and Jar were seeded at a cell density of 3×10^4^ cells. They were allowed to reach confluence at 37°C in 5% CO_2_. A single vertical “scratch” wound was made using a sterile P200 tip from the top to bottom of each well, running through the horizontal line. Cells were washed twice with PBS to remove cell debris and 500 µL serum free cell growth medium was added to each well. The wound area of each well just above and just below the horizontal line was imaged using a DMI8 inverted light microscope, along with tile scan coordinates available with AxioVision Rel. 4.8 software.

Following baseline measurement, serum-free media were replaced with 500 µL MSC-conditioned medium and prescribed cultivation media or serum free media (pre-equilibrated in 21% or 1% O_2_). Plates were incubated at 37°C in 21% or 1% O_2_ for 24 h before wound areas were reimaged. The area of the wound site at baseline and at 24 h was measured using ImageJ v1.48. The values obtained were used to calculate the percentage of wound closure over 24 h.

### Real-time polymerase chain reaction

Ribonucleic acid (RNA) from placental samples was extracted using Qiagen RNeasy kit (Qiagen, Hilden, Germany) according to the manufacturer’s instructions. Cell-line RNA was extracted using TRIzol™ reagent (Thermo Fisher Scientific, UK) as previously described (17) and reverse transcribed using custom made master mix (Thermo Fisher Scientific, UK). Complementary deoxyribonucleic acid was prepared for real-time quantitative polymerase chain reaction (qPCR) using the Roche Lightcycler Probes 480 Master kit (Roche, USA) and Roche Realtime Ready TaqMan gene expression mono hydrolysis probes for human FKBPL, CD44, and 18S (Roche, USA). The resulting crossing points were calculated using the Roche LightCycler 480 software and quantified using the standard curve efficiency. Sample crossing point values were corrected to 18S expression. Data analysis was carried using the ΔΔCT method.

### Western blotting

Alongside migration and invasion assays, BeWo, Jar, HUVEC, and ACH-3P cell lysates were harvested using radioimmunoprecipitation assay buffer (SantaCruz Biotechnology, USA) supplemented with protease and phosphatase inhibitor cocktails (Roche, UK). Cell lysates were reduced in 4× and 10× Bolt LDS Sample buffer (ThermoFisher, UK) and subjected to Western blotting. Blots were probed with specific primary antibody and appropriate HRP-linked secondary immunoglobulin G (Cell signaling, UK) at 1:10 000. The membrane was imaged with G:Box (SynGene, India) after incubation using either West Dura or Femto chemiluminescent (ThermoFisher, UK). Antibodies CD44H (R&D Systems, cat: BBA10); FKBPL (Proteintech, USA cat: 10060-1-AP); anti-glyceraldehyde 3-phosphate dehydrogenase (GAPDH) (cell signaling, UK, cat: G9545); all antibodies were used at 1:1000 unless otherwise stated.

### Statistical analysis

The data were analyzed using a 2-tailed *t*-test or 1-way or 2-way analysis of variance (ANOVA), followed by Sidak’s or Tukey’s multiple comparisons tests and adjusted *P* values reported. Values were considered statistically significant if *P* < 0.05. Experiments involving cell lines and primary cells included the results from a minimum of 3 independent repeats, and the results were expressed as the mean ± the standard error of the mean (SEM). Analysis was performed using Prism 5 software (GraphPad Software, La Jolla, CA, USA). The statistical analyses of the results in relation to FKBPL, CD44, and the clinical characteristics of Cohort 3, the SCOPE Cork subset (33) were performed using SPSS 20.0 (IBM, New York, USA). After initial testing for normality (Shapiro-Wilks test), differences between groups (preeclampsia *vs* controls) were tested using independent samples *t*-test or Mann-Whitney, where appropriate. Preeclampsia and control samples were matched for age and BMI. Receiver operating characteristic (ROC) curve analysis was used to obtain cut-off values for primary variables (CD44/FKBPL ratio and MAP) measured at 20 weeks gestation. Participants were stratified into either a group with low levels (below the cut-off) or a group with high levels (above the cut-off value) of the parameters in question. The contribution of the CD44/FKBPL ratio to the risk of preeclampsia development was assessed using binary logistic regression, which included age, BMI, weight change and MAP as the possible confounders. Multinomial logistic regression was used to test the combined effect of the CD44/FKBPL ratio and MAP on the risk of developing preeclampsia. Significance was set at *P* < 0.05.

### Study approvals

For all human samples used in this study, written informed consent was received from participants prior to inclusion in the study. Studies involving human samples were also approved by local institutional ethics review boards. All personal details were anonymized, and participants were identified by unique identifying numbers, not by names.

## Results

### The diagnostic potential of the FKBPL-CD44 pathway after the onset of clinical preeclampsia

**Figure 1.**
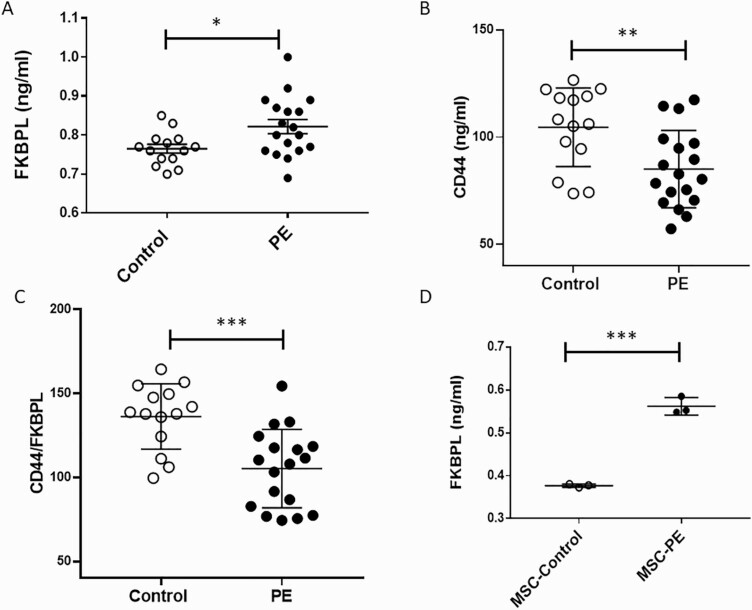
Secreted FKBPL and CD44 from plasma and primary mesenchymal stem cells are increased and decreased, respectively, in preeclampsia. (**A**) Plasma FKBPL is increased in pregnant women with preeclampsia (n = 18) compared to controls (n = 14); 2-tailed unpaired *t*-test. (**B**) Plasma CD44 is decreased in pregnant women with preeclampsia (n = 18) compared to controls (n = 14); 2-tailed unpaired t-test. (**C**) CD44/FKBPL ratio is decreased in pregnant women with preeclampsia (n = 18) compared to controls (n = 14); 2-tailed unpaired *t*-test. (**D**) Secreted FKBPL concentration was higher in cultured MSCs isolated from abdominal fat tissue in preeclampsia (n = 3) vs. controls (n = 3). FKBPL secretion in preeclampsia was adjusted to the number of MSC; 2-tailed unpaired t-test. Data are presented as mean ± SEM. *P < 0.05; ***P < 0.001.

Using patients’ samples from the Cohort 1 (Table 1) following clinical diagnosis of preeclampsia, we measured plasma FKBPL and CD44 concentration (n = 18, preeclampsia; n = 14, control) as well as secreted FKBPL from MSCs isolated from pregnant women with preeclampsia (n = 3) and normotensive controls (n = 3). Plasma FKBPL concentration was increased in women with preeclampsia compared to controls (Fig. 1A; 0.81 ng/mL ± 0.018 SEM *vs* 0.76 ng/mL ± 0.011 SEM, *P* < 0.05) whereas plasma CD44 concentration was decreased in the same samples (Fig. 1B; 85.1 ng/mL ± 4.2 SEM *vs* 104.7 ng/mL ± 4.9 SEM, *P* < 0.01). The CD44/FKBPL ratio was also significantly reduced in preeclampsia cases compared to controls (Fig. 1C; 105.3 ng/mL ± 5.5 SEM *vs* 136.3 ng/mL ± 5.2, *P* < 0.001). Statistical significance was maintained (*P* = 0.02) when the CD44/FKBPL ratio was adjusted for differences in gestational age between the preeclampsia and control groups (Table 1), using logistic regression. A cut-off value based on the ROC curve (area under the curve [AUC] = 0.84, *P* = 0.001) of less than 134.6 (sensitivity 94.4 and specificity 71.4) was associated with a 3.3-fold increase in likelihood ratio. There were no differences in age (*P* = 0.65), BMI (*P* = 0.34), or gravidity (*P* = 0.74) between groups; however, gestational age was lower (*P* = 0.001) in the preeclampsia group (Table 1).

**Table 1.** Clinical characteristics of normotensive pregnancies and pregnancies with established preeclampsia (Cohort 1)

	Normotensive (n = 14)	Preeclampsia (N = 18)	*P*-value
Age (year)	31.77 ± 2.20	32.23 ± 3.79	0.66
BMI (kg/m^2^)	30.97 ± 6.59	34.01 ± 4.98	0.16
Gestational age	39.00 ± 2.77	34.50 ± 2.71	**0.001**
Gravidity	1.5 (1-5)	2 (1-1)	0.73
Term pregnancies	1.5 (1-3)	1 (0-4)	0.11
Preterm pregnancies	0 (0-1)	1 (0-2)	**0.016**
Previous abortions	0 (0-3)	0 (0-1)	0.29
Number of living children	1.5 (1-3)	1.5 (1-5)	0.77

Age, BMI, and gestational age are presented as mean ± SD; all other values are presented as median with min-max range. Student *t*-test and Fischer test. Bolded P-values indicates a significant difference between the two groups.

Abbreviation. BMI, body mass index.

Similarly, secretion of FKBPL from adipose-tissue isolated MSCs from women with preeclampsia was increased compared to controls (0.562 ng/mL ± 0.012 SEM *vs* 0.377 ng/mL ± 0.002 SEM; *P* < 0.001; Fig. 1D). The 2 groups of women (controls *vs* preeclampsia) were matched for age (26.7 ± 3 *vs* 27 ± 7, *P* = 0.8), BMI (36.6 ± 6.2 *vs* 32.3 ± 3.0, *P* = 0.7) and gestational age (38.1 ± 0.85 *vs* 32.6 ± 5.1, *P* = 0.1).

**Figure 2.**
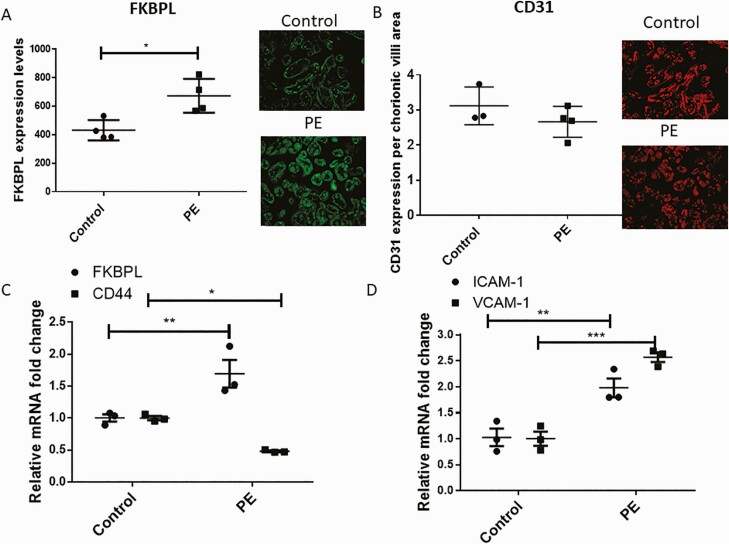
FKBPL expression is increased concomitantly with CD44 downregulation and VCAM-1 and ICAM-1 upregulation in preeclampsia. Immunofluorescence staining of placentae from women with and without preeclampsia (n = 4) was performed using an anti-FKBPL rabbit anti-human polyclonal primary antibody **(A)** at a 1:150 dilution and an anti-CD31 guinea pig anti-human polyclonal primary antibody **(B)** at 1:50. Four images per slide were taken at 20× magnification and the mean fluorescence quantified using Image J (2-tailed unpaired *t*-test, n = 4). RNA was extracted from the frozen placental tissue, converted to complementary deoxyribonucleic acid before FKBPL and CD44 **(C)** or VCAM-1 and ICAM-1 **(D)** mRNA expression was measured using qPCR. The mRNA expression was adjusted to 18S and to healthy controls (n = 3; 2-way ANOVA with Sidak’s multiple comparison test). Data are presented as mean ± SEM. *P < 0.05, **P < 0.01, and ***P < 0.001. Abbreviation: PE, preeclampsia.

In light of FKBPL’s critical role in developmental, physiological, and pathological angiogenesis as an anti-angiogenic protein (18) via CD44 (17), we investigated both FKBPL and CD44 expression in placental samples from women with and without preeclampsia (Cohort 2, Table 2). Placental expression of FKBPL, CD44, intercellular adhesion molecule (ICAM), and vascular cell adhesion molecule (VCAM) from women with preeclampsia were compared to their normotensive controls (matched for age, BMI, and gestational age; Table 2). The expression of FKBPL was observed in both syncytiotrophoblasts and blood vessels (Fig. 2A pictures inset). Our results demonstrated increased FKBPL protein expression (>1.5-fold) in preeclampsia cases compared to controls (Fig. 2A; *P* < 0.05, n = 4). The endothelial cell marker, CD31, was not significantly different between preeclampsia and control samples (Fig. 2B; *P* = 0.27; n = 4 [preeclampsia] and n = 3 [control]). At the messenger RNA (mRNA) level, using RNA lysates from the same placental samples, we confirmed increased FKBPL mRNA expression and a concomitant reduction in its target gene, CD44 (Fig. 2C; FKBPL: *P* < 0.01; CD44: *P* < 0.05; n = 3). The presence of endothelial dysfunction in preeclampsia has been well established (34), which was confirmed in our cohort by increased expressions of the endothelial dysfunction markers, VCAM-1 and ICAM-1 (Fig. 2D; VCAM-1: *P* < 0.01; ICAM-1: *P* ˂ 0.001; n = 3).

**Table 2.** Maternal baseline characteristics from women whose placenta was collected (Cohort 2)

	Preeclampsia (n = 4)	Control (n = 4)	*P* value
BMI	32.0 ± 7.6	29.7 ± 2.8	0.38
Age	34.0 ± 4.3	31.8 ± 6.2	0.42
Gestational week (weeks ± days)	36.7 ± 1.8	37.9 ± 2.6	0.36
First trimester right hand sBP	130.8 ± 3.6	125.1 ± 7.7	0.17
First trimester right hand dBP	90.6 ± 13.9	73.5 ± 22.3	0.18
First trimester left hand sBP	133.8 ± 6.6	125.1 ± 9.8	0.07
First trimester left hand dBP	91.3 ± 10.2	81.4 ± 8.7	0.20
The highest recorded BP	170.25 ± 13.05	134.75 ± 4.27	0.007
Fetus gender (male)	3	3	1.00

Values are presented as mean ± SD.

Abbreviations: dBP, diastolic blood pressure; sBP, systolic blood pressure.

### The predictive potential of the FKBPL-CD44 pathway before the onset of clinical preeclampsia

Having shown that FKBPL is regulated by established preeclampsia, we wanted to investigate the predictive biomarker potential of FKBPL and CD44 using plasma samples from nulliparous women with singleton pregnancies at 15 and 20 weeks of gestation from Cohort 3 (Table 3) (33). There was no difference in CD44 plasma concentration (115.9 ± 24.5 standard deviation [SD], n = 60 *vs* 121.7 ± 28.7 SD, n = 60; *P* = 0.23). However, the FKBPL plasma concentrations were significantly lower (0.72 [0.52-1.10], n = 61 *vs* 1.0 [0.59-1.30], n = 59; *P* = 0.03), at 15 weeks of gestation in women who proceeded to develop preeclampsia. At 20 weeks of gestation, both CD44 (127.5 ng/mL ± 30.2 SD, n = 62 *vs* 115.3 ng/mL ± 26.4 SD, n = 60, *P* = 0.02) and FKBPL plasma concentrations (0.65 [0.44-1.08], n = 57 *vs* 0.89 [0.61-1.16], n = 58; *P* = 0.01) were higher and lower in the preeclampsia group compared to controls, respectively. When CD44 and FKBPL were combined as the CD44/FKBPL ratio, the differences between preeclampsia and control groups demonstrated an increase in the plasma CD44/FKBPL ratio (15th week: 143.1 [113.2-259.2], n = 57, 128.8 [91.2-176.1], n = 56; *P* = 0.02; 20th week: 181.1 [121.3-302.4], n = 54 *vs* 143.1 [101.3-176.3], n = 55; *P* = 0.004, Table 3).

**Table 3.** Clinical characteristics of pregnant women at 15 and 20 weeks of pregnancy (Cohort 3)

Variable	15 Weeks of Pregnancy			20 Weeks of Pregnancy		
	Controls	Cases	*P*-value	Controls	Cases	*P*-value
Age (years)	30.1 ± 3.6	30.2 ± 4.2	0.95	30.1 ± 3.6	30.2 ± 4.2	0.95
BMI (kg/m^2^)	25.8 ± 4.0	26.9 ± 5.0	0.16	n/a	n/a	n/a
Gestational age at delivery (weeks)	38.2 ± 13.3	37.9 ± 13.3	0.9	38.2 ± 13.3	37.9 ± 13.3	0.9
Weight change (kg)	n/a	n/a	n/a	3.2 ± 1.9	2.9 ± 2.0	0.97
sBP (mmHg)	103.2 ± 8.9	109.8 ± 10.5	**<0.001**	107.7 ± 10.3	112.1 ± 10.5	**0.02**
dBP (mmHg)	64.9 ± 6.6	69.1 ± 7.1	**<0.001**	66.3 ± 6.2	70.1 ± 7.6	**0.002**
MAPB (mmHg)	77.7 ± 6.8	82.6 ± 7.7	**<0.001**	80.1 ± 7.1	84.1 ± 8.0	**0.003**
Random glucose (mmol/l)	5.1 ± 0.9	5.5 ± 1.2	**0.049**	5.4 ± 1.1	5.8 ± 1.0	**0.047**
CD44 (ng/mL)	115.9 ± 24.5	121.7 ± 28.7	0.23	115.3 ± 26.4	127.5 ± 30.2	**0.020**
FKBPL (ng/mL)	1.0 [0.59–1.30]	0.72 [0.52–1.10]	**0.034**	0.89 [0.61–1.16]	0.65 [0.44–1.08]	**0.011**
CD44/FKBPL ratio	128.8 [91.2–176.1]	143.1 [113.2–259.2]	**0.018**	143.1 [101.3–176.3]	181.1 [121.3–302.4]	**0.004**

All values are presented as mean ± SD or median with interquartile range. Two-tailed unpaired t-test (normalized distribution) or Man-Whitney (non-normalized distribution). All clinical characteristics were available for 128 samples in each group at 15/20 gestation (preeclampsia and healthy controls); FKBPL analysis was successful for 117 controls and 118 cases; CD44 analysis was successful for 120 controls and 122 cases. Bolded P-values indicate a significant difference between the two groups.

Abbreviations: BMI, body mass index; cases, preeclampsia; dBP, diastolic blood pressure; MAP, mean arterial blood pressure, sBP, systolic blood pressure.

Based on the ROC curve analysis, we were able to determine a suitable cut-off point for the CD44/FKBPL ratio of 143.6 (AUC = 0.659, *P* = 0.004; sensitivity = 0.7, specificity = 0.51) or 155.1 (sensitivity = 0.6 and specificity = 0.6) for prediction of the risk of preeclampsia at 20 weeks of gestation. Using this cut-off point, a univariate logistic regression model demonstrated, that women with a CD44/FKBPL ratio above 143.6 or 155.1 had a 2.5- or 2.4-fold increased risk of developing preeclampsia later in pregnancy (*P* = 0.02, *P* = 0.03), respectively. Multivariate logistic regression models using BMI, age, weight change, and MAP as confounders, demonstrated that the CD44/FKBPL ratio persisted to be associated with preeclampsia independently of BMI, age, weight change, and MAP (143.6: odds ratio [OR] = 2.3 95% confidence interval [CI] 1.03-5.2, *P* = 0.04; 155.1: OR = 2.3 95% CI 1.05-5.2, *P* = 0.04; Tables 4 and 5). The combined effect of the CD44/FKBPL ratio and MAP (cut-off = 82.5 mmHg; AUC = 0.648, *P* = 0.004; sensitivity = 0.62, specificity = 0.59), on the risk of developing preeclampsia was investigated using multinomial logistic regression models. Participants were divided into 4 groups: (i) low-risk (reference) group—women with a low plasma CD44/FKBPL ratio (˂143.6 or 155.1) and MAP (˂82.5 mmHg), (ii) high-risk group (CD44/FKBPL > 143.6 or 155.1 and MAP > 82.5 mmHg), (iii) the medium risk 1 group included pregnancies with a high CD44/FKBPL and low MAP, and (iv) the medium risk 2 group included pregnancies with a low CD44/FKBPL ratio and high MAP (Table 6). Statistical significance was confined to the high-risk model where we demonstrated a 3.9-fold (143.6: 95% CI 1.3-11.8, *P* = 0.016) or 4.1-fold (155.1: 95 CI 1.4-12.4, *P* = 0.013) increased risk of developing preeclampsia (Table 6).

**Table 4.** CD44/FKBPL ratio (>143.6) at 20 weeks gestation is an independent risk factor for developing preeclampsia

CD44/FKBPL ratio at 20 weeks gestation	Controls	Cases	OR (95% CI)	*P*-value
CD44/FKBPL ratio < 143.6	28 (51)	16 (30)	1.0^a^	
CD44/FKBPL ratio > 143.6	27 (49)	38 (70)	2.32 (1.03-5.23)	0.043
Clinical characteristics				
BMI (kg/m^2^)	25.78 ± 4.01	26.93 ± 5.03	1.01 (0.91-1.10)	0.900
Age (years)	30.14 ± 3.58	30.18 ± 4.16	0.98 (0.87-1.09)	0.649
Weight change (kg)	3.19 ± 1.92	2.87 ± 2.00	0.90 (0.74-1.10)	0.319
MAP (mmHg)	80.13 ± 7.10	84.13 ± 8.01	0.97 (0.91-1.03)	0.334

CD44/FKBPL ratios are given as *n* (%) for both cases and controls. All clinical characteristics are given as mean  ± SD for both cases and controls.

Abbreviations: BMI, body mass index; CI, confidence interval, MAP, mean arterial pressure; OR, odds ratio.

^a^Reference category.

**Table 5.** CD44/FKBPL ratio (>155.10) at 20 weeks of gestation is an independent risk factor for developing preeclampsia

CD44/FKBPL ratio at 20 weeks gestation	Controls	Cases	OR (95% CI)	*P*-value
CD44/FKBPL ratio < 155.10	33 (60)	21 (39)	1.0^a^	
CD44/FKBPL ratio > 155.10	22 (40)	33 (61)	2.33 (1.05–5.17)	**0.038**
Clinical characteristics				
BMI (kg/m^2^)	25.78 ± 4.01	26.93 ± 5.03	0.97 (0.88–1.07)	0.531
Age (years)	30.14 ± 3.58	30.18 ± 4.16	0.94 (0.85–1.05)	0.279
Weight change (kg)	3.19 ± 1.92	2.87 ± 2.00	0.91 (0.75–1.11)	0.354
MAP (mmHg)	80.13 ± 7.10	84.13 ± 8.01	0.99 (0.94–1.05)	0.876

CD44/FKBPL ratios are given as *n* (%) for both cases and controls. All clinical characteristics are given as mean  ± SD for both cases and controls. Bolded P-values indicate a significant difference between the two groups.

Abbreviations: BMI, body mass index; CI, confidence interval; MAP, mean arterial pressure; OR, odds ratio.

^a^Reference category.

**Table 6.** Odds ratio for developing preeclampsia based on the combination of MAP and the CD44/FKBPL ratio (cut-off 143.6 or 155.1) at 20 weeks of gestation

MAP and CD44/FKBPL	Controls, *n* (%)		Cases, *n* (%)		OR (95% CI)		*P*-value	
	143.6	155.1	143.6	155.1	143.6	155.1	143.6	155.1
Low risk^a^	16 (29)	18 (33)	7 (13)	8 (15)	1.0	1.0		
Medium risk 1^b^	13 (24)	11 (20)	14 (26)	13 (24)	2.46 (0.78-7.90)	2.66 (0.84-8.46)	0.130	0.098
Medium risk 2^c^	12 (22)	15 (27)	9 (17)	13 (24)	1.71 (0.49-5.92)	1.95 (0.64-5.95)	0.394	0.241
High risk^d^	14 (25)	11 (20)	24 (44)	20 (37)	3.92 (1.30-11.84)	4.09 (1.35-12.43)	**0.016**	**0.013**

Cut off value for CD44/FKBPL ratio (143.6 or 155.1) and MAP (82.5 mmHg) were determined based on the ROC analysis. Bolded P-values indicate a significant difference between the two groups.

Abbreviations: CI, confidence interval; OR, odds ratio.

^a^Low risk: Low CD44/FKBPL and low MAP (reference category).

^b^Medium risk 1: High CD44/FKBPL and low MAP.

^c^Medium risk 2: Low CD44/FKBPL and high MAP.

^d^High risk: High CD44/FKBPL and high MAP.

### The role of FKBPL and CD44 proteins in placental development

**Figure 3.**
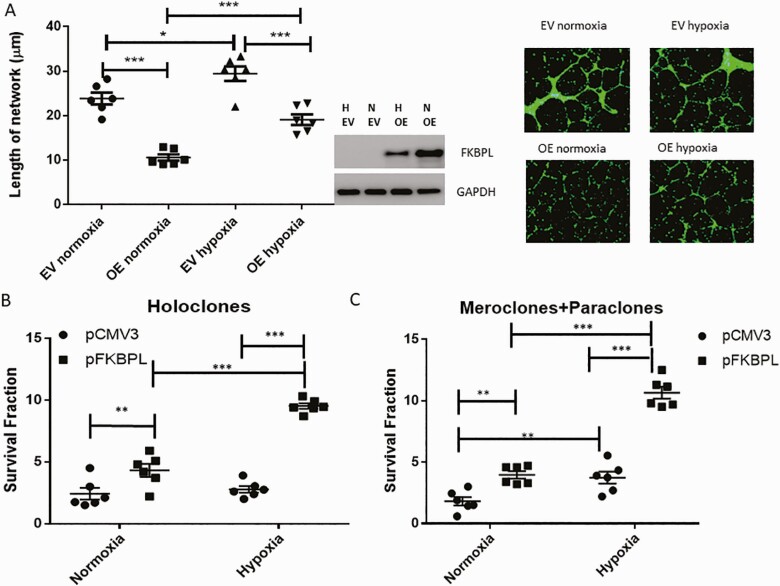
FKBPL restores hypoxia-mediated angiogenic effects, and increases the colony formation in trophoblasts. **(A)** FKBPL was overexpressed using 1μg pFKBPL or an EV (pCMV3) plasmid in HUVECs for 24 h, cells were stained with calcein and trypsinized before plating 50 000 cells in Matrigel and exposing them to hypoxia (1%) or normoxia (21%) for 24 h. Cells were imaged using a Leica DMi8 fluorescent microscope and the tubules quantified using ImageJ software. Representative pictures are shown in the inset. **(B and C)** BeWo were seeded overnight before being transfected with 1μg pFKBPL or an EV (pCMV3) control plasmid for 24 h. Following transfection, 1000 and 2000 cells were seeded in 6 well plates, allowed to adhere overnight, and incubated for 16 to 18 days in 21% or 1% O_2_ before being fixed in crystal violet 0.4% (w/v) in methanol (n = 6). Statistical analyses were performed using 1-way ANOVA with Tukey’s or Sidak’s multiple comparisons tests; n = 6. *P < 0.05, **P < 0.01, ***P < 0.001. Abbreviations: EV, empty vector; H, hypoxia; OE, overexpression; N, normoxia; VC, vehicle control.

Endothelial dysfunction and inappropriate SUA remodeling by trophoblasts have been closely linked to inadequate placentation and the pathogenesis of preeclampsia (35). To test the functional role of FKBPL in angiogenesis important for appropriate placental development, we transiently overexpressed FKBPL in HUVECs. Overexpression of FKBPL compared to empty vector (EV) resulted in a reduction of the length of tubule networks in normoxia (*P* < 0.001, n = 6; Fig. 3A). As expected, hypoxic conditions demonstrated a pro-angiogenic effect, leading to an increase in the length of tubule networks (*P* = 0.024, n = 6; Fig. 3A, representative pictures inset), while FKBPL overexpression in hypoxia ameliorated this effect, restoring angiogenesis to normal (*P* < 0.001, n = 6; Fig. 3A). This suggests that FKBPL has a key role in hypoxia-mediated effects on angiogenesis.

When we investigated the functional effect of FKBPL on the trophoblast clonogenic potential, important for SUA remodeling, we showed that transient overexpression of FKBPL compared to EV increased the number of holoclones (Fig. 3B, *P* = 0.005 [normoxia 21% O_2_], *P* < 0.001 [hypoxia 1% O_2_], n = 6) resembling stem cells, and the number of meroclones and paraclones resembling more differentiated cells (36) (Fig. 3C, *P* = 0.005 [normoxia 21% O_2_], *P* < 0.001 [hypoxia 1% O_2_], n = 6) (37), in hypoxia or normoxia. No effect of hypoxia was demonstrated on BeWo holoclones (Fig. 3B), whereas an increase in the numbers of meroclones and paraclones were observed between normoxia and hypoxia following transient transfection with an EV control plasmid (Fig. 3C, *P* < 0.001, n = 6).

### FKBPL and CD44 as targets of emerging mesenchymal stem cell treatment for preeclampsia

**Figure 4.**
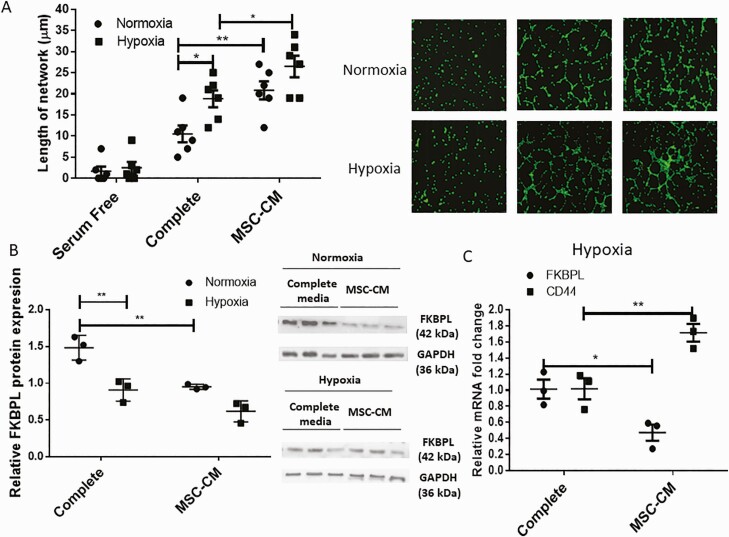
Angiogenic properties of HUVECs are improved as a result of hypoxia and mesenchymal stem cell treatment, in association with reduced FKBPL and increased CD44 expression. (**A**) HUVEC differentiation was evaluated using a tubule formation assay; cells were stained with calcein and trypsinized before plating 50 000 cells in Matrigel and exposing them to hypoxia (1% O_2_) or normoxia (21% O_2_) for 6 h in complete or MSC-CM medium (n = 6). Cells were imaged using a Leica DMi8 fluorescent microscope and the tubules quantified using ImageJ software. Representative pictures are shown in the inset. (**B**) Protein lysates were collected and Western blotting performed to determine FKBPL (42 kDa) and glyceraldehyde 3-phosphate dehydrogenase (37kDa) protein expressions (n = 3). FKBPL protein expression was quantified using ImageJ and adjusted to glyceraldehyde 3-phosphate dehydrogenase expression. A representative picture of the Western blot is shown in the inset. (**C**) Real-time qPCR analyses of FKBPL and CD44 mRNA expression relative to the 18S housekeeping gene were carried out using HUVECs exposed to complete or MSC-CM in hypoxia for 24 h (n = 3). Results are expressed as mean ± SEM; statistical analyses were performed using 2-way ANOVA with Sidak’s or Tukey’s multiple comparisons test. *P < 0.05, **P < 0.01. Abbreviation: MSC-CM, mesenchymal stem cell–conditioned medium.

Currently there are no effective therapeutic strategies for preeclampsia. However, a number of treatments including MSC-based therapies are emerging in this setting (38). We therefore, investigated the functional role of MSCs in angiogenesis and trophoblast migration important for appropriate placental development. More specifically, the influence of hypoxia and MSC-conditioned medium (MSC-CM) on endothelial cell tubule formation and trophoblast migration, as well as the effect on FKBPL signaling, was determined. Hypoxia (1%) led to an increase in the length of the tubule network of the HUVECs (*P* = 0.014, n = 6). MSC-CM treatment also led to an increase in the length of the tubule networks compared to normal or complete medium both under normoxic (*P* = 0.002, n = 6) and hypoxic conditions (*P* = 0.02, n = 6; Fig. 4A), 6 h after seeding the cells in Matrigel. Overall, improved angiogenic properties were associated with reduced FKBPL protein expression following exposure to hypoxia in complete medium conditions (*P* < 0.01, n = 6) and between complete medium and MSC-CM conditions in normoxia (*P* < 0.01, n = 6; Fig. 4B). In hypoxia, at the mRNA level, FKBPL was reduced (*P* = 0.021, n = 3) and CD44 concomitantly increased (*P* = 0.005, n = 3) as a result of MSC-CM treatment of HUVECs (Fig. 4C). No differences in VCAM-1 and ICAM-1 mRNA expressions were observed between complete medium and MSC-CM in normoxia or hypoxia.

**Figure 5.**
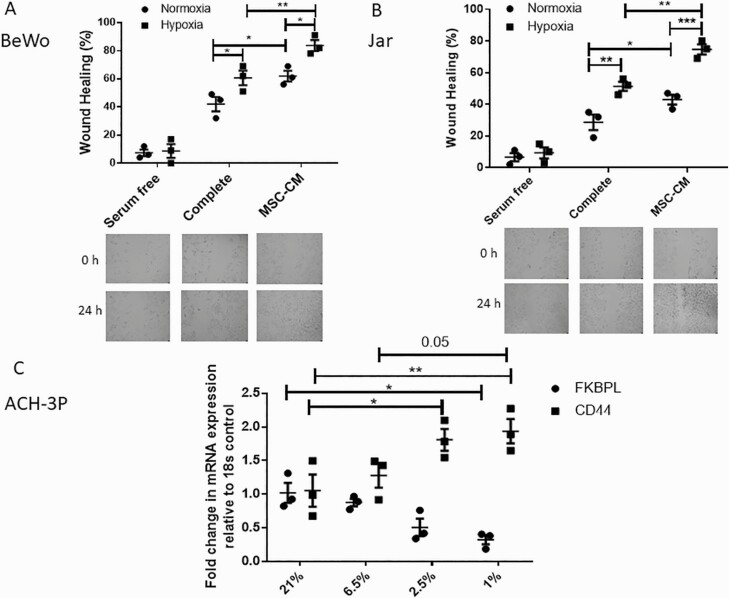
Trophoblast cell migration is stimulated by hypoxia or MSC treatment and hypoxia regulates FKBPL and CD44. Migration of BeWo (**A**) or Jar (**B**) cells was evaluated using a wound healing scratch assay, in hypoxia (1% O_2_) or normoxia (21% O_2_), treated with complete medium or MSC-CM for 24 h (n = 3). (**C**) Real-time qPCR analyses of FKBPL and CD44 mRNA expression (n = 3) relative to the 18S housekeeping gene were performed in ACH-3P trophoblast cells. Results are expressed as mean ± SEM; statistical analyses were performed using 2-way ANOVA with Sidak’s or Tukey’s multiple comparisons test. *P < 0.05, **P < 0.01, and ***P < 0.001. Abbreviation: MSC-CM, mesenchymal stem cell–conditioned medium.

When trophoblast cells, BeWo and Jar, were exposed to hypoxia and/or MSC-CM, an increase in cell migration was also observed in both complete medium and MSC-CM as a result of hypoxia (BeWo: *P* = 0.03 [complete; n = 3], *P* = 0.01 [MSC-CM, n = 3]; Jar: *P* = 0.002 [complete, n = 3], *P* < 0.001 [MSC-CM, n = 3]; Fig. 5A and 5B), and MSCs in normoxia (BeWo: *P* = 0.02, n = 3; Jar: *P* = 0.04, n = 3; Fig 5A and 5B) or hypoxia (BeWo: *P* = 0.008, n = 3; Jar: *P* = 0.001, n = 3; Fig 4A and 4B).

Furthermore, we used a custom made first trimester extravillous and endovascular trophoblast cell line, ACH-3P cells, which more closely resembles primary first trimester trophoblasts (30,39). We exposed ACH-3P cells to varying concentrations of oxygen (21%, 6.5%, 2.5%, and 1%), similar to what is observed in the first trimester during placental development (40,41), except for the 21% oxygen, which is above physiological levels, but extensively utilized in cell culture experimentation as a control. At the mRNA level, similar to previously described studies, FKBPL and CD44 expression showed opposite trends at lower oxygen concentrations (FKBPL: *P* = 0.037 [21% *vs* 1%]; CD44: *P* = 0.021 [21% *vs* 2.5%], *P* = 0.006 [21% *vs* 1%]; Fig. 5C).

These data suggest that hypoxia and MSCs have a pro-angiogenic effect on both endothelial and trophoblast cells and that the mechanism targets the novel anti-angiogenic FKBPL-CD44 pathway. This suggest that MSCs, driven by their secretome, could be further explored as a potential novel treatment for preeclampsia.

## Discussion

Currently, reliable biomarkers are lacking that can stratify women at high risk of developing late-onset preeclampsia or evolving preeclampsia between the second and third trimester to enable better monitoring and early detection of this dangerous condition. Controlled angiogenesis is necessary for appropriate placental development during pregnancy and any aberrant changes in the angiogenic balance are closely associated with preeclampsia (42).

**Figure 6.**
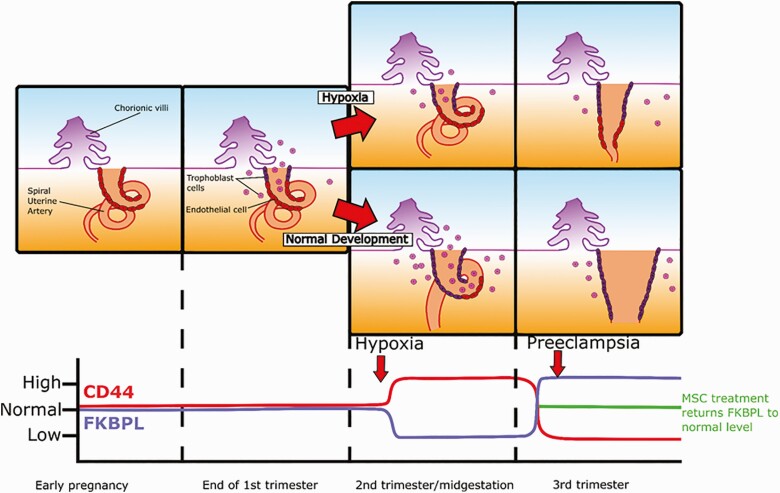
Summary of FKBPL and CD44 changes throughout gestation in pregnancies complicated by preeclampsia. In early embryogenesis a loose connection of chorionic projections, the apposition, implants into the endometrium allowing for the subsequent development of the placenta. Trophoblast cells (in red) migrate from the chorionic villi and start the process of spiral uterine artery remodeling replacing endothelial cells (in purple). FKBPL and CD44 levels starts to decrease and increase, respectively, at the beginning of the second trimester as a result of continuous hypoxia and likely compensatory pro-angiogenesis in pregnant women who proceed to develop preeclampsia. Toward the end of the second trimester or the beginning of the third trimester, the levels start to change in the opposite direction, likely just before the onset of preeclampsia indicating anti-angiogenic state. MSC therapy can then be employed to restore the angiogenic balance and treat preeclampsia.

This is the first study to demonstrate an important and novel role for the anti-angiogenic pathway, FKBPL-CD44, in the pathogenesis of preeclampsia, which could be utilized for the prediction, diagnosis, and treatment of preeclampsia. Our results show that the CD44/FKBPL ratio is associated with the risk of preeclampsia independently of established risk factors including age, BMI, MAP, and weight gain (33,43,44), from 20 weeks of gestation. Plasma FKBPL concentration was reduced at both 15 and 20 weeks of gestation whereas plasma CD44 concentration was increased at 20 weeks of gestation in a low-risk cohort of women who proceeded to develop preeclampsia. High CD44 and low FKBPL are likely driven by hypoxia, which is a pro-angiogenic stimulus (45), as a result of potential compensatory angiogenesis early in pregnancy. At 20 weeks of gestation, a high CD44/FKBPL ratio and high MAP were associated with an approximately 4-fold increased risk of preeclampsia. The early predictive value of clinical parameters such as maternal blood pressure in combination with angiogenic biomarkers has been highlighted previously, however mainly in preterm preeclampsia (43,46). The vast majority of the cases in our cohort proceeded to develop late-onset preeclampsia. Interestingly, in established preeclampsia, the CD44/FKBPL ratio showed an opposite pattern, suggesting that these potential longitudinal changes in CD44/FKBPL ratio from trimester 2 to 3 could reflect evolving preeclampsia and, therefore, could be explored for early diagnosis (Fig. 6). These findings in relation to a reduced the CD44/FKBPL ratio in established preeclampsia reflective of restricted angiogenesis, align with previous reports of a preeclampsia-related anti-angiogenic state where the sFlt-1/sEng ratio is increased and the vascular endothelial growth factor/PlGF ratio is decreased (47-49).

In addition to demonstrating the early biomarker potential of FKBPL and CD44, we also demonstrated the key roles for these novel proteins in trophoblast and endothelial cell function using in vitro models of SUAs remodeling important for placental development and healthy pregnancy. The importance of inappropriate response to low oxygen sensing by trophoblast cells has been previously linked with preeclampsia (50). Our results demonstrate hypoxia-mediated downregulation of FKBPL expression, as well as associated functional changes including migration, tubule formation, and clonogenic potential—all key features in placental development. Notably, hypoxia as a pro-angiogenic stimulus, which is present in the ischemic placenta in preeclampsia (51), led to a reduction in endothelial and trophoblast FKBPL protein and mRNA expressions and a concomitant increase in CD44 mRNA expression. These changes reflected those observed in patient plasma samples at 15 and 20 weeks of gestation in pregnant women, a whole trimester before the onset of preeclampsia. Our previous work in *Fkbpl*^*+/-*^ knockdown transgenic mice indeed showed a pro-angiogenic phenotype; however, these mice demonstrated early signs of endothelial dysfunction (18). Therefore, a hypoxia-induced pro-angiogenic effect that drives FKBPL levels down could lead to endothelial dysfunction and hence the development of preeclampsia. Our in vitro data presented here with HUVECs following overexpression of FKBPL in hypoxia restored angiogenesis to normal levels likely preventing this endothelial dysfunction.

MSCs have shown therapeutic potential in various diseases due to their pro-angiogenic, anti-inflammatory, and low immunogenicity profiles. Accumulating evidence suggests that their beneficial effects are exerted through their secretome; a previous study has demonstrated that MSC-conditioned media is capable of restoring angiogenic balance in *ex vivo* models of preeclampsia by reducing sFlt-1 (52). FKBPL has not been implicated previously in MSC-mediated pro-angiogenic and anti-inflammatory mechanisms of action. Recently MSCs were shown to be effective in reducing symptoms (ie, blood pressure and proteinuria) of preeclampsia using in vivo models of preeclampsia, mainly by targeting inflammatory mechanisms (14,15). In our study, we showed that MSCs are capable of enhancing migration of trophoblasts, in both hypoxia and normoxia, as well as tubule formation of endothelial cells, in association with reduced levels of the anti-angiogenic protein, FKBPL, and increased pro-angiogenic CD44 mRNA expression. In our recent study, we demonstrated that in addition to inhibiting angiogenesis, FKBPL is also involved in regulating a number of inflammatory pathways including signal transducer and activator of transcription 3 (20), which has been implicated in preeclampsia and trophoblast functionality (53,54). Therefore, these results suggest that MSCs could be utilized in established or clinically diagnosed preeclampsia to restore FKBPL levels to normal, potentially improving the vascular response and symptoms of preeclampsia.

While this is the first study that implicates the FKBPL-CD44 pathway in preeclampsia, there are some limitations including the small number of placental samples obtained from the Cohort 2. However, the trend in FKBPL/CD44 expression levels is the same in placentae from Cohort 2 and plasma samples from Cohort 1, both of which are postdiagnosis of preeclampsia. The most promising prediction value of the CD44/FKBPL ratio is at week 20 of gestation, which is later than needed for administration of aspirin as a preventative therapy (ie, before week 16) (55). However, aspirin is only effective at preventing the risk of early-onset preeclampsia, and the majority of the patients from the Cohort 3 proceeded to develop late-onset preeclampsia. Although we did not have longitudinal samples from the same study before and after the clinical onset of preeclampsia, using separate cohorts of pregnant women, we demonstrated that the CD44/FKBPL ratio shows opposite patterns of secretion in plasma postdiagnosis (reflective of restricted angiogenesis) compared to prediagnosis (reflective of stimulated angiogenesis, which could be compensatory at this stage of gestation). This could be beneficial for risk stratification of women with evolving preeclampsia and for early detection. In the future, CD44 and FKBPL plasma concentrations should be investigated in a larger cohort of patients using longitudinal plasma samples from all three trimesters and postdiagnosis of preeclampsia.

In summary, we have identified a new angiogenesis-related pathway, FKBPL-CD44, with an important role in the pathogenesis of preeclampsia. Both FKBPL and CD44 are regulated by hypoxia and appear to have key roles in the processes important for SUA remodeling preceding the onset of preeclampsia. This has both potential diagnostic and therapeutic applications for preeclampsia, particularly in relation to emerging MSC-based treatment.

## Conclusion

The plasma CD44/FKBPL ratio could provide a novel risk stratification approach for preeclampsia at 20 weeks of gestation, capable of identifying women at high risk, who otherwise appear healthy. The CD44/FKBPL ratio could also identify the cases of evolving preeclampsia through longitudinal changes potentially leading to early detection of this condition. Similarly, the anti-angiogenic FKBPL-CD44 axis is inhibited as part of the therapeutic mechanism of MSCs, which are currently in the preclinical stage of development for the treatment of preeclampsia.

## Data Availability

The datasets generated and/or analyzed during the current study are not publicly available but are available from the corresponding author upon reasonable request.

## Additional Information

***Disclosure Summary:*** LM and TR are listed as inventors on the FKBPL patent (“Assay method for determining the risk of preeclampsia”; WO2018051125A1). The authors have no conflicting financial interests.
